# Supplementary material for: Epigenetic gene expression links heart failure to memory impairment
Source: EMBO Mol Med. 2021 Jan 20;13(3):e11900. doi: 10.15252/emmm.201911900 (PMC7933944; doi:10.15252/emmm.201911900)
Supplement: Supplementary file 1 — Appendix [file EMMM-13-e11900-s001.docx]

**Appendix**

**Table of content**

Page 2: Appendix figure S1 and its legend

Page 3: Appendix figure S2 and its legend

**Appendix Fig S1**


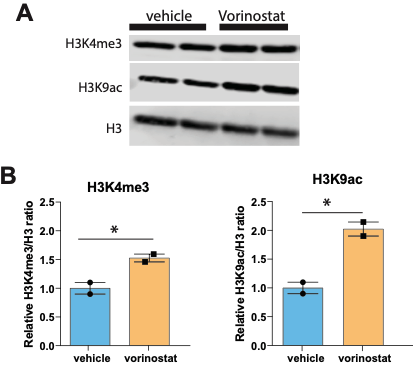


**Appendix Fig S1. Vorinostat increases H3K4me3 level in primary neurons.** Primary mouse hippocampal neuronal cultures were treated for 1h with Vorinostat (1µM) or vehicle before proteins were isolated and subjected to immunoblot analysis. A) Western blot for H3K4me3 and H3K9ac after 1hour of SAHA (1 uM) and vehicle treatment. B) Semi-quantitative analysis of immunblot analysis. Two replicates represent pooled samples (n = 3) from two independent experiments. Experiment was done at day in vitro 10. Relative Intensity was normalized to H3 level. The data reveal that Vorinostat treatment significantly increases bulk levels of H3K4me3 and H3K9ac. **P* < 0.05, unpaired t-test; two-tailed, Error bars indicate mean ± sem. Please note that in addition to these data, we also provide evidence that Vorinostat-treatment affects hippocampal H3K4me3 in neurons when measured via ChIP-sequencing Please see Fig. 5 G.

**Appendix Fig S2**


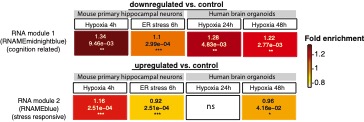


**Appendix Fig S2: Hypogeometric overlap analysis comparing conserved the gene-expression networks of RNA module 1 and 2 to hypoxic and ER stress conditions.** Heatmaps summarizing results from hypergeometric tests for genes in RNA module 1 and 2 with stress conditions in different experimental settings. Hypoxia (1% O2, 4h) and endoplasmic stress (tunicamycin 2 ug/mL, 6h) was modeled in primary hippocampal neurons (See Fig 1). Gene expression data on hypoxia from human brain organoid data was retrieved from Pasca et al, 2019. Up and down regulated genes (FDR<0.05) were determined by comparing to corresponding controls. Enrichment significance cutoff: FDR < 0.05. Color intensity represents fold enrichment.
