# Supplementary material for: Epigenetic gene expression links heart failure to memory impairment
Source: EMBO Mol Med. 2021 Jan 20;13(3):e11900. doi: 10.15252/emmm.201911900 (PMC7933944; doi:10.15252/emmm.201911900)
Supplement: Supplementary file 2 — Expanded View Figures PDF [file EMMM-13-e11900-s002.pdf]

Expanded View Figures

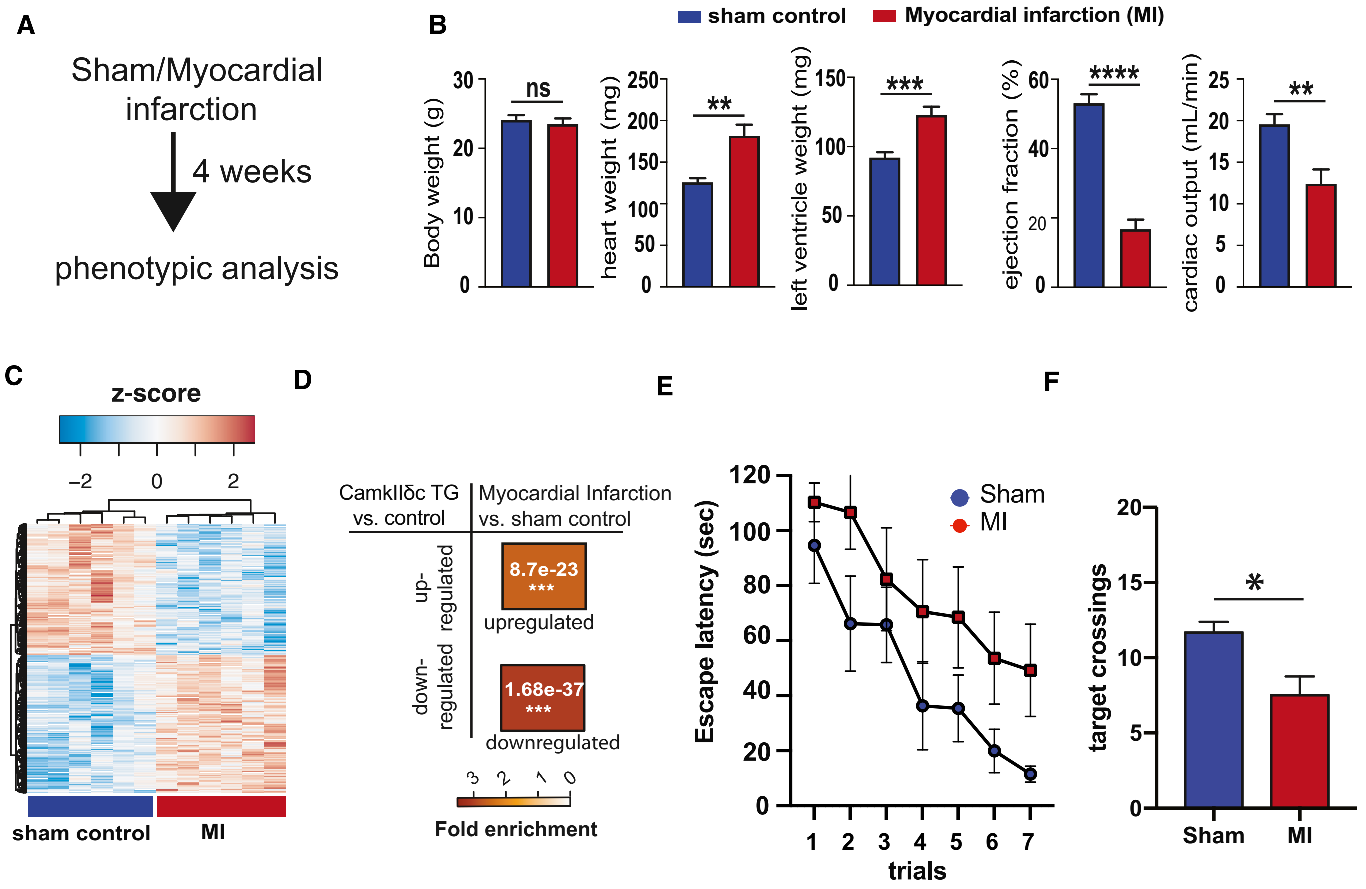

Figure EV1. Hippocampal gene expression and memory function is impaired in a mouse models for myocardial infarction (MI).

A Experimental design. 2-month-old male C57BL6/J mice were operated to induce myocardial infarction through coronary artery ligation using an established protocol (Mohamed *et al*, 2018). Sham-operated mice were used as controls. Phenotypic analysis was performed 4 weeks later when mice were 3 month of age.

B MI mice exhibited no difference in body weight (left panel), while heart and left ventricle weight were significantly increased when compared to the sham control group. Ejection fraction and cardiac output were significantly impaired in MI mice (right panels). Number of mice (Sham = 9, MI = 8). unpaired *t*-test, two-tailed $^{**}P < 0.01$, $^{***}P < 0.001$, $^{****}P < 0.0001$.

C RNA sequencing was performed from the hippocampal CA1 region of 3-month-old MI and sham control mice ($n = 6$/group). The heatmap shows significantly deregulated genes (down-regulated: 517, up-regulated: 542, adjusted P value < 0.05). Red color represents up-regulated genes, while the blue color shows the down-regulated genes.

D Hypergeometric overlap analysis comparing the up- and down-regulated genes detected in the hippocampal CA1 region of CamkIIδc TG mice to the genes deregulated in hippocampus of MI mice compared to sham control group. Please note that there was a highly significant overlap (FDR < 0.05), suggesting that similar hippocampal genes are affected in response to MI and CamkIIδc overexpression-mediated heart failure. Fisher's hypergeometric test, Benjamini–Hochberg (BH) correction.

E Four weeks after surgery MI and sham control mice were subjected to the Barnes maze to assay hippocampus-dependent spatial memory. The escape latency was significantly impaired in MI mice, when compared to the sham control group indicating impaired learning behavior (Sham = 9, MI = 8; Mixed-effects analysis).

F Bar chart showing the number of target crossings during the memory retrieval test performed after the last day of training. MI mice performed significantly worse when compared to the sham control group. Number of replicates (Sham = 9, MI = 8). Two-tailed, unpaired *t*-test, $^{*}P < 0.05$.

Data information: Bars and error bars indicate mean ± SEM.

 © 2021 The Authors

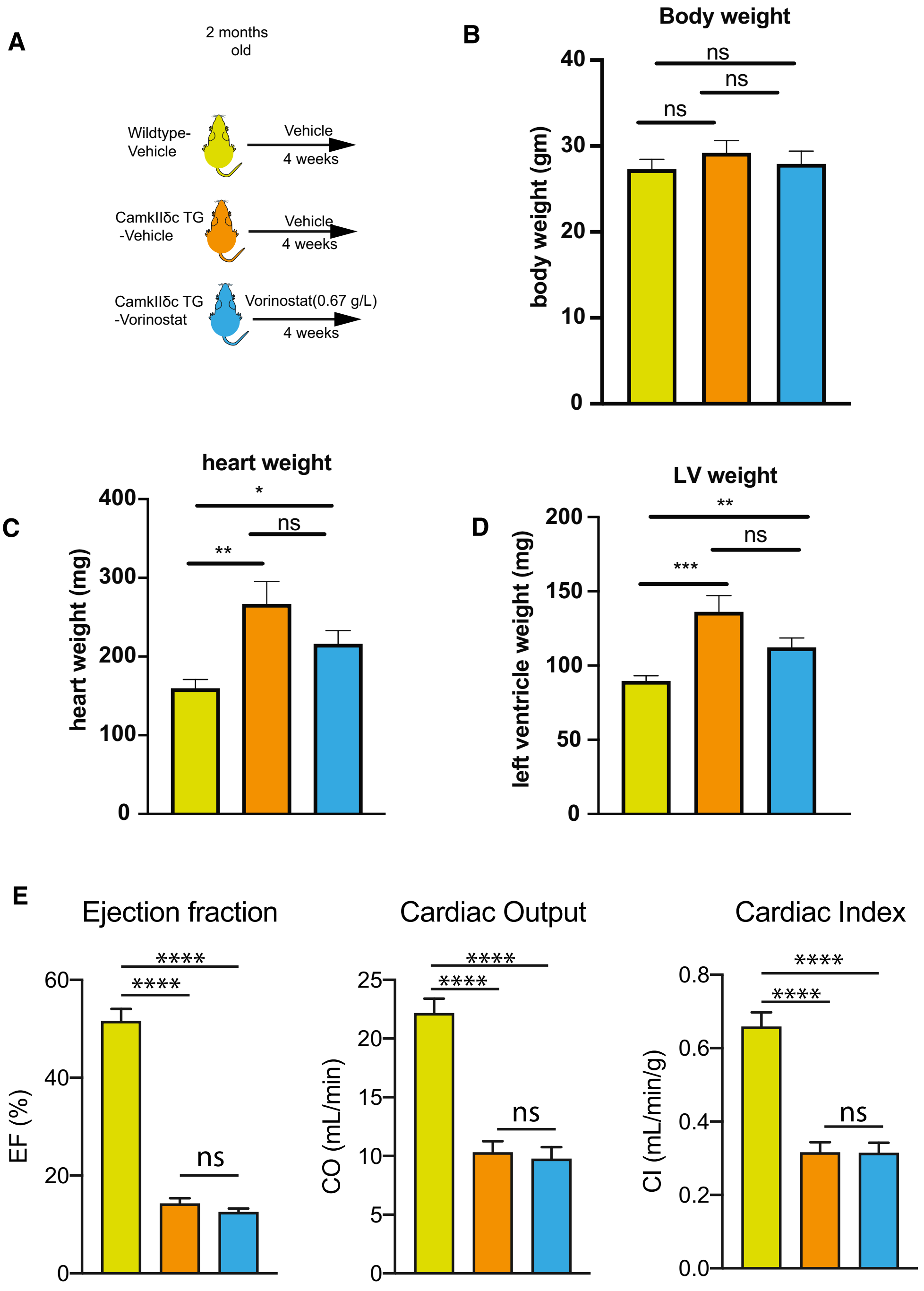

Figure EV2. Cardiac function is not significantly affected in CamkIIδc TG mice upon Vorinostat treatment.

A Scheme of the experimental design. Vorinostat treat was initiated at 2 month of age and analysis was performed at 3 month of age. $n = 12$/group.

B Bar chart showing that body weight was similar among groups. $n = 12$/group. One-way ANOVA.

C, D Bar chart showing that heart weight (C) and left ventricle (LV) weight (D) was increased in vehicle and Vorinostat-treated CamkIIδc TG mice when compared to the wild-type vehicle control group. $n = 12$/group. $^*P < 0.05$, $^{**}P < 0.01$, $^{***}P < 0.001$. Kruskal–Wallis test.

E Bar charts showing that ejection fraction (left panel), cardiac output (CO; middle panel), and cardiac index (CI, right panel) is significantly impaired in vehicle-treated CamkIIδc TG mice ($n = 12$) when compared to corresponding control ($n = 12$) group. These impairments were not affected by Vorinostat treatment. $^{****}P < 0.0001$. ns, not significant, Kruskal–Wallis test.

Data information: Bars and error bars indicate mean ± SEM.

© 2021 The Authors

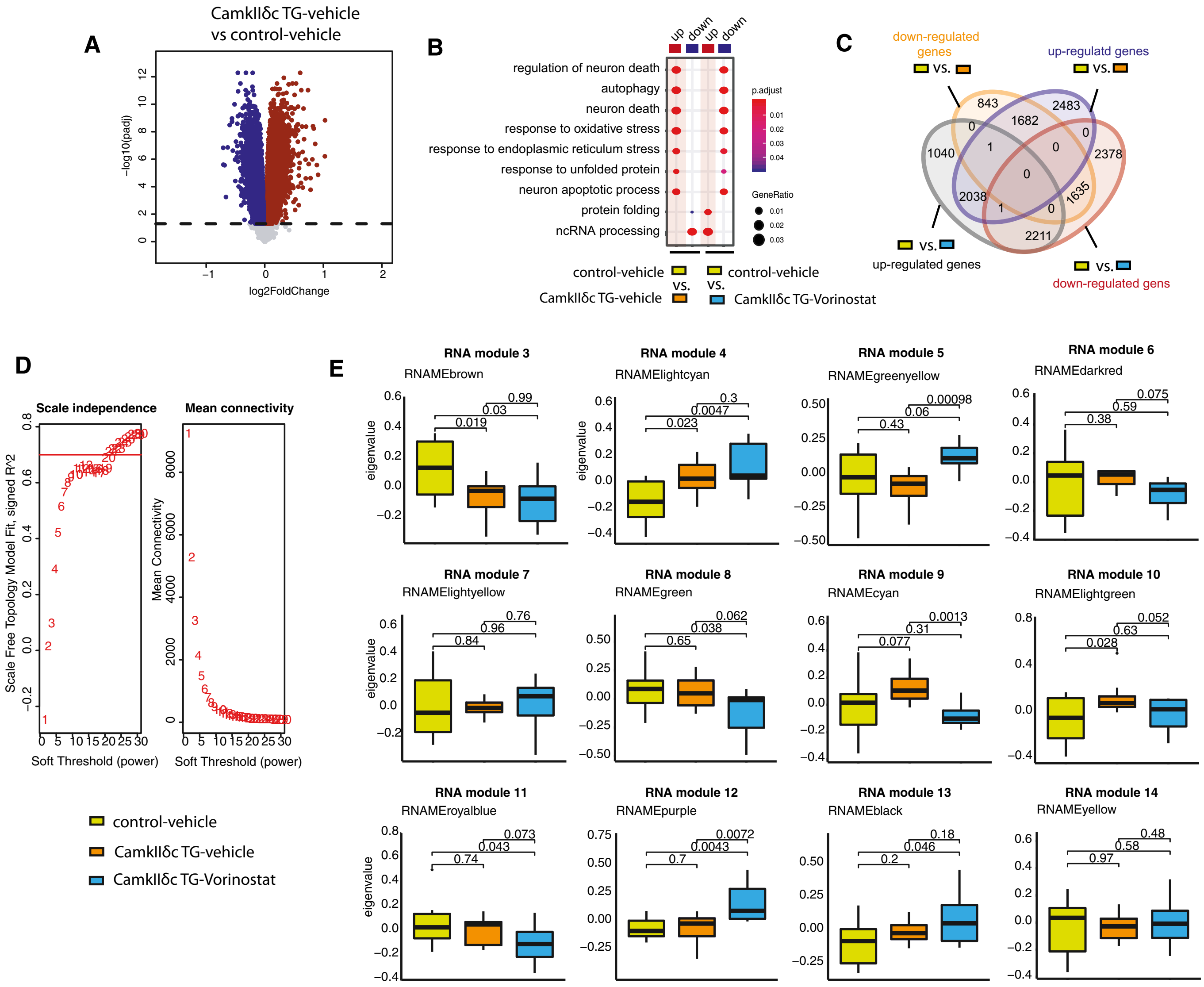

Figure EV3. Weighted gene co-expression analysis upon Vorinostat treatment.

- A Volcano plot showing significantly deregulated genes in the hippocampal CA1 region, when comparing vehicle-treated CamkIIδc Tg mice to vehicle-treated control mice (FDR < 0.05). Up- and down-regulated genes are represented in dark red and dark blue colors, respectively.
- B Pathways affected in the hippocampal CA1 region when comparing vehicle-treated wild-type vs CamkIIδc mice to Vorinostat-treated CamkIIδc vs vehicle-treated wild-type mice. Note that Vorinostat treatment ameliorates pathways affected in CamkIIδc TG mice for pathway increased and decreased under pathological conditions.
- C Venn diagram showing common and uniquely deregulated genes between groups.
- D Soft power selection based on scale independence and mean connectivity for different modules identification in WGCNA.
- E Different modules representing distinct expression patterns among groups. Y-axis representing Eigen expression of given cluster/module. Wild-type vehicle: $n = 10$; CamkIIδc TG vehicle $n = 9$; CamkIIδc TG-Vorinostat $n = 9$. Kruskal–Wallis test. The center band indicates the median, while the upper and lower band represent the 75th and 25th percentiles, respectively. The whiskers represent the smallest and largest values in the 1.5× interquartile range.

 © 2021 The Authors

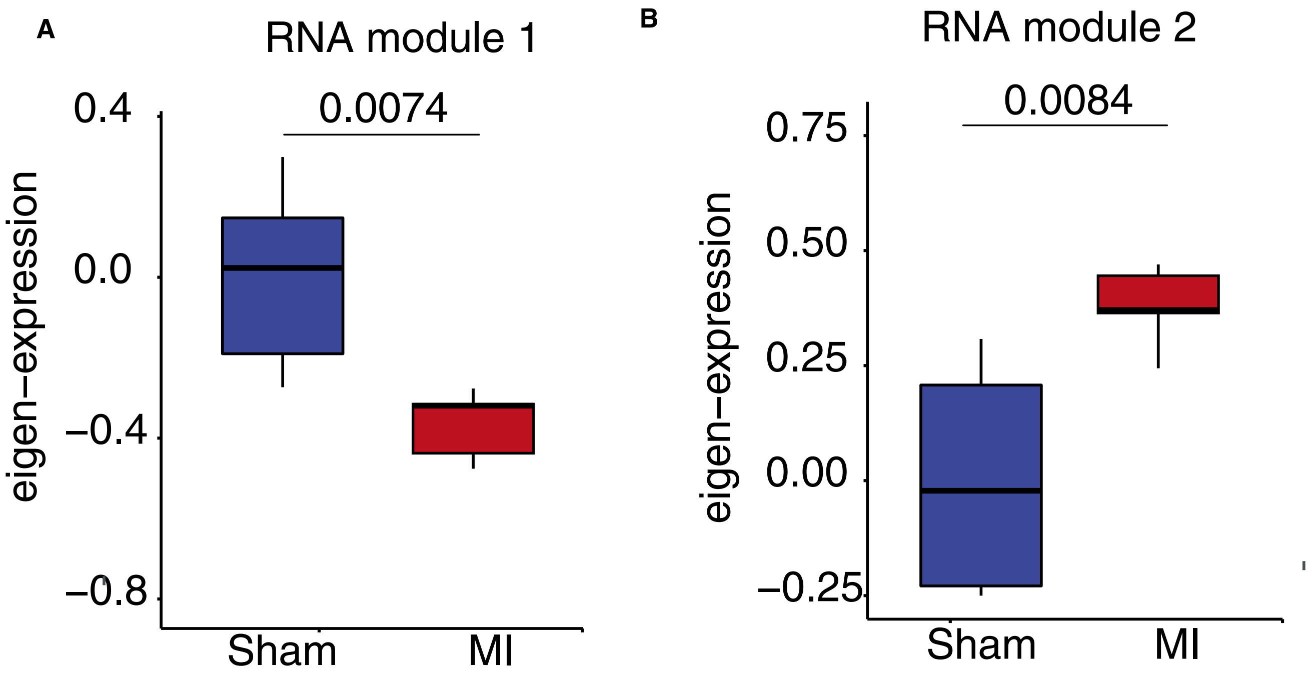

Figure EV4. Co-expression of RNA module 1 and RNA module 2 in Sham and MI mice.

Cluster genes retrieved from CamkIIIδc mice were used to calculate eigenvalue from RNA-seq data generated in Sham and MI operated mice and plotted. $N = 6$ per group. In Fig A and B, the upper and lower band represent the 75th and 25th percentiles, respectively, while the center line indicates the median. The whiskers represent the smallest and largest values in the 1.5× interquartile range.

A Expression of RNA module 1. $n = 6$ per group, Wilcoxon test.
B Expression of RNA module 2. $n = 6$ per group, Wilcoxon test.

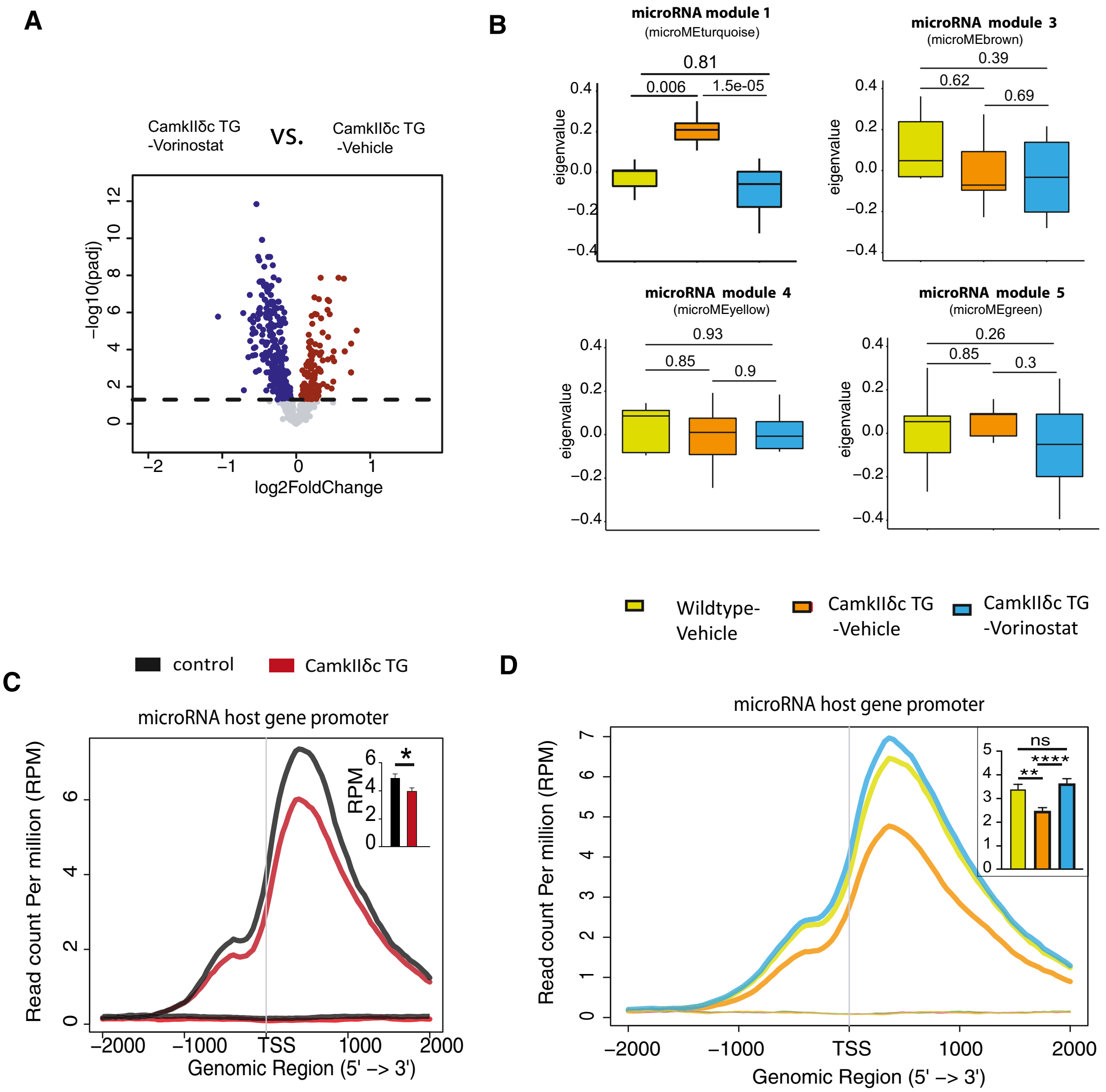

Figure EV5. Vorinostat-induced microRNA expression changes in the hippocampal CA1 region of CamkIIδc TG mice.

A Volcano plot showing differentially expressed microRNAs (FDR < 0.05) when comparing Vorinostat-treated to vehicle-treated CamkIIδc TG mice.

B Expression of microRNA modules from WGCNA analysis in three experimental groups. Wild-type vehicle: $n = 9$; CamkIIδc TG vehicle $n = 7$; CamkIIδc TG-Vorinostat $n = 10$; Kruskal–Wallis test. In boxplot, the upper and lower band represent the third and first quartiles, respectively, while the center line indicates the median. The whiskers represent the smallest and largest values in the 1.5× interquartile range.

C H3K4me3 profile at promoter of genes that harbor microRNAs of microRNA module 2 (See Fig 5F). H3K4me3 level at the TSS of these genes is significantly reduced in CamkIIδc TG mice when compared to control mice. Inset shows statistical analysis; unpaired *t*-test, two-tailed, *$P < 0.05$. Bars and error bars indicate mean ± SEM. Number of replicates for H3K4me3 ChIP profile analysis (Wild-type vehicle: 3; CamkIIδc TG vehicle $n = 4$; CamkIIδc TG-Vorinostat $n = 4$).

D Comparison of H3K4me3 profile at the TSS of genes that harbor microRNAs of microRNA module 2 (same as panel C) among wild-type Vehicle, CamkIIδc TG vehicle and CamkIIδc TG-Vorinostat groups. Average H3K4me3 profile at promoter of microRNA host genes is down-regulated in CamkIIδc TG vehicle, but restored in CamkIIδc TG-Vorinostat mice. One-way ANOVA; **$P < 0.01$, ****$P < 0.0001$. Bars and error bars indicate mean ± SEM. Number of replicates for analyzed H3K4me3 profile (Wild-type vehicle: 3; CamkIIδc TG vehicle $n = 4$; CamkIIδc TG-Vorinostat $n = 4$).

 © 2021 The Authors
